# RNA activation in insects: From emerging evidence to molecular applications

**DOI:** 10.1111/imb.70058

**Published:** 2026-07-09

**Authors:** Sassan Asgari

**Affiliations:** ^1^ School of the Environment and School of Chemistry and Molecular Biosciences The University of Queensland Brisbane Queensland Australia

**Keywords:** gene expression, insects, RNA activation, RNA biology, RNA interference, RNAa, RNAi

## Abstract

RNA interference is commonly used as a reverse genetics tool to investigate gene function. In insects, this is achieved by introducing double‐stranded RNA or synthetic short‐interfering RNAs (siRNAs). However, RNA activation (RNAa) is less frequently utilised in these studies. RNAa can be achieved by targeting a gene's promoter with antisense RNA, which triggers endogenous transcription. Unlike the siRNA mechanism, which is well characterised, the RNAa mechanism is less well known. In the literature, ‘RNAa’ has been adopted to describe diverse small RNA‐mediated upregulation mechanisms that may not necessarily function through the canonical RNAa mechanism described in mammals, which involves interaction of the small RNA with the promoter region. Although the outcomes of RNAi and RNAa differ, some components are shared between their pathways, such as Argonaute proteins. In this minireview, RNAa, its canonical and non‐canonical mechanisms, and applications in insect research are examined with the aim of encouraging its use as a tool in Insect Molecular Biology.

## Graphical abstract

- Canonical RNA activation involves small RNAs interacting with promoter sequences, but there is also evidence of non‐canonical RNAa in eukaryotes.
- Emerging evidence suggests the presence of RNAa in insects.
- RNAa can be utilised as a tool for functional genomic studies in insects.

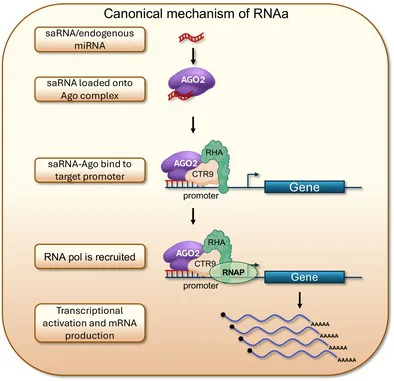

## INTRODUCTION

RNA‐based technologies have revolutionised Insect Molecular Biology research over the past 20 years. Among these, RNA interference (RNAi) has become the most commonly used method for studying gene function and functional genomics. From an applied perspective, RNAi has also gained considerable attention as an environmentally friendly alternative to synthetic chemical pesticides. This is primarily due to its species specificity, which makes it safe for non‐target and beneficial insects, as well as for humans and animals (reviewed in Narva et al., 2025). RNAi is initiated by the presence of double‐stranded RNA, whether endogenous or exogenous, which triggers the production of short interfering RNAs (siRNAs) through the ribonuclease activity of the Dicer 2 protein (reviewed in Hussain et al., 2016). These siRNAs then induce the formation of the RNA‐induced silencing complex (RISC), in which the Argonaute 2 (AGO2) protein plays a central role by facilitating binding to target RNA sequences guided by the loaded siRNAs. The interaction between siRNA and its target is sequence‐specific, relying on complete complementarity between the siRNA and target sequences.

In contrast, the idea that short RNAs can *activate* gene expression, known as RNA activation (RNAa), has received limited attention in insect research. In canonical RNAa, small activating RNAs (saRNAs) bind to a gene's promoter region and trigger upregulation. This phenomenon was first observed in mammalian cells (Janowski et al., 2007; Li et al., 2006), leading to a paradigm shift in how small RNAs are viewed in transcriptional regulation, from mere silencers to activators as well. This phenomenon was later demonstrated in plants (Shibuya et al., 2009), *Caenorhabditis elegans* (Seth et al., 2013; Turner et al., 2014), insects (De Hayr et al., 2020) and ticks (Kwofie et al., 2023); although some of these may not strictly follow the canonical RNAa. In addition to saRNAs, endogenous microRNAs (miRNAs) can also induce gene expression by binding to the promoter region. This is referred to as miRNA‐mediated RNAa (mi‐RNAa) (reviewed in Qian et al., 2025). For example, hsa‐miR‐373 induced E‐cadherin expression in human PC3 cells by binding to complementary sequences in its promoter region (Place et al., 2008). miRNAs may also mediate upregulation mechanisms by binding to the mRNA sequences of a target gene (see mi‐RNAa in insects). miRNAs are short non‐coding RNAs produced from coding or non‐coding mRNA transcripts that contain stem‐loop structures (Bartel, 2009). The stem‐loop is then cleaved by Drosha in the nucleus and then transported into the cytoplasm, where the hairpin head is removed by Dicer‐1, inducing the formation of miRNA‐RISC complex in which AGO1 plays an instrumental role. Unlike siRNAs, miRNAs do not require complete sequence complementarity to their target sequences to be functional. Evidence of positive transcriptional regulation by miRNAs also changed views of miRNAs as solely negative regulators, to include them also as RNA activators (Guo et al., 2014; Qian et al., 2025).

Results from gain‐of‐function experiments can also be valuable in insect functional genomics. These include knock‐ins via CRISPR, transgenesis and inducible plasmid expression systems. However, these methods are often technically demanding and time‐consuming. Conversely, RNAa could provide a complementary, non‐transgenic approach to temporarily enhance endogenous gene expression. Despite this, it has been somewhat underutilised in Insect Molecular Biology research. This minireview aims to highlight RNAa to the research community, summarising its molecular mechanisms, reviewing emerging evidence for RNAa in insects and exploring its potential applications in Insect Molecular Biology, pest management and vector biology. Further, instances of RNAa in its broad sense are also discussed in this review, which do not necessarily utilise the canonical RNAa mechanism of gene induction but may have potential applications for gene induction in insect studies.

## MOLECULAR BASIS OF RNA ACTIVATION

Unlike RNAi, the mechanism of action of RNAa is not well understood, especially in insects. This may have caused hesitations in adopting the tool. A key difference between RNAi and canonical RNAa is that, unlike siRNAs, which guide target RNA cleavage in the cytoplasm, saRNAs localise to the nucleus where they interact with the transcriptional machinery to activate gene expression. However, similar to RNAi, RNAa depends on AGO proteins (Chu et al., 2010; Li et al., 2006; Portnoy et al., 2011), although most research has been conducted in mammalian cells. It seems that the saRNA‐loaded AGO protein, transported from the cytoplasm to the nucleus, helps assemble an RNA‐induced transcriptional activation (RITA) complex and enables its interaction with chromatin at target sites by allowing RITA to engage with RNA polymerase II and start transcription (Figure 1). RITA consists of an AGO protein (usually AGO2), RNA helicase A (RHA), Cln Three requiring 9 (CTR9) and other proteins. RHA likely helps unwind the DNA helix to enable saRNA binding. CTR9 is part of the PAF1 complex, which participates in transcription and regulates RNA polymerase II activity (Francette et al., 2021). This process is also associated with monoubiquitination of histone 2B (Portnoy et al., 2016). Another study showed that saRNAs promote nucleosome removal in the region between the RNA polymerase II binding site and the transcription start site, thereby facilitating assembly of the transcription preinitiation complex and activating transcription (Wang & Hu, 2017). These findings suggest RNAa involves epigenetic modifications. In the case of mi‐RNAa, for instance, miR‐34a, the miRNA guides RITA to the paused RNA polymerase II, leading to transcription initiation (Ohno et al., 2022).

**FIGURE 1:**
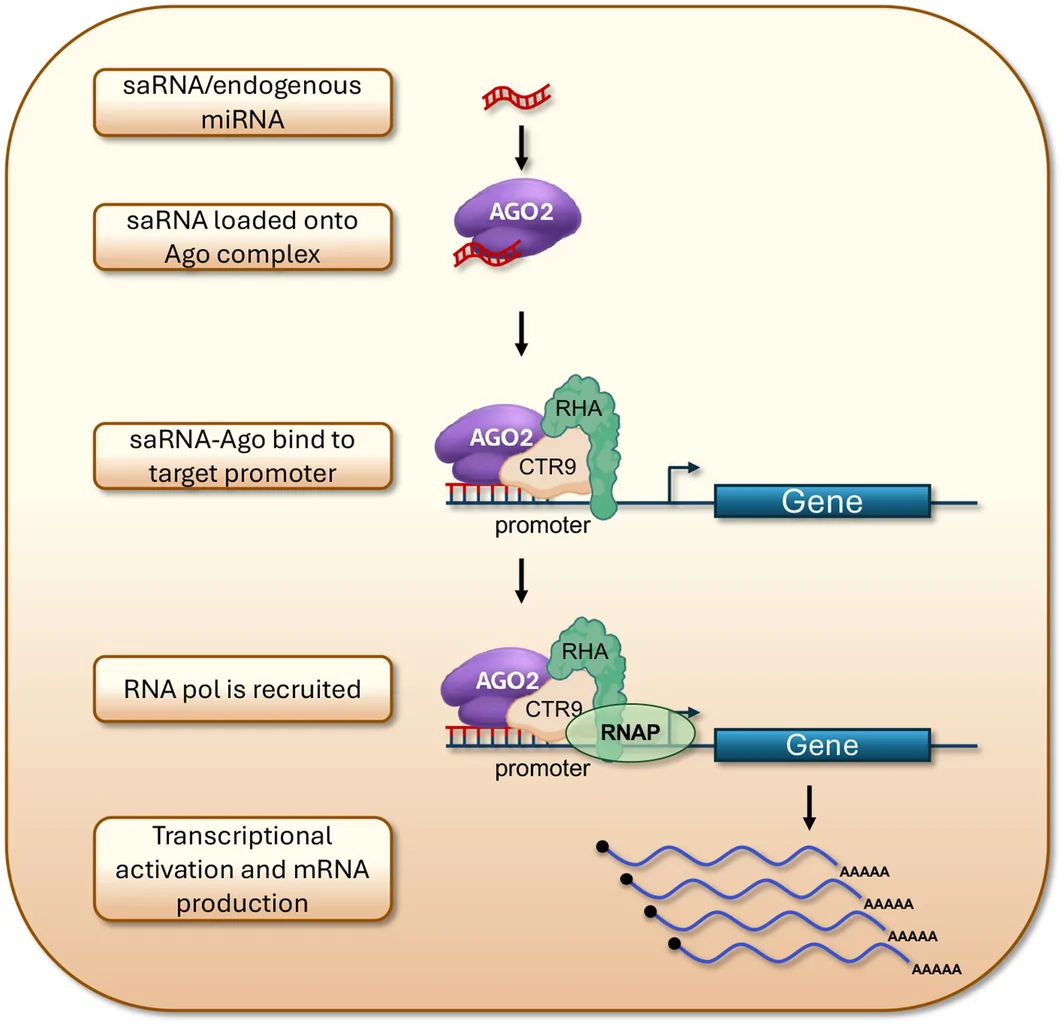
RNA activation via small RNAs. Short activating RNA (saRNA) duplex or endogenous miRNA is loaded onto Argonaute 2 (AGO2) protein, which will guide the single‐stranded RNA in association with other proteins (RHA and CTR9) to the promoter region on the genomic DNA, leading to the recruitment of transcription factors and RNA polymerase (RNAP), activating transcription. Biorender and PowerPoint were used to generate this figure.

In the case of saRNAs, the antisense strand appears to be the main functional arm associated with AGO2 in mammalian cells (Meng et al., 2016). Similar to miRNAs, the seed region (nucleotides 2–8 from the 5′ end of the small RNA) is crucial for saRNA function through complementarity to target sequences. Therefore, mutations in the seed region may abolish saRNA function (Meng et al., 2016). Further, mutations in the target genomic DNA sequences eliminate RNAa, highlighting the importance of the specificity of the target sequences (Meng et al., 2016). In addition to interacting with the promoter region, saRNAs may also target the antisense transcripts to a gene's promoter, leading to RNAa. It is suggested that when saRNA binds to the antisense transcript at a promoter, it can cause cleavage of these transcripts and thus eliminate their repressive effect on the gene (reviewed in Wang & Hu, 2017).

Based on studies in mammalian cells, the effect of RNAa can persist for more than 10 days, extending to 3–4 weeks in some instances (Wang et al., 2015), further suggesting the involvement of epigenetic mechanisms. Although the onset of RNAa could be delayed, starting 3 days after the application (Place et al., 2010).

While RNAa has been reported from *C. elegans*, evidence for the involvement of the specific components of the RITA complex has not been provided. lin‐4 is a miRNA that regulates stem cell fate in *C. elegans* by regulating lin‐14 and lin‐28 developmental genes (Ambros, 2000). Evidence suggests that lin‐4 binds to a conserved lin‐4‐complementary element (LCE) in its promoter region and activates its expression, involving recruitment of RNA polymerase II. An LCE binding protein complex was identified, but its composition was not determined (Turner et al., 2014). In another form of RNAa, in its broad sense, in *C. elegans*, the CSR‐1 AGO protein binds 22G‐RNAs, which guide AGO to cognate nascent transcripts, enabling interactions with RNA polymerase II and local chromatin. This interaction results in the recruitment of histone‐modifying enzymes, promoting epigenetic activation. 22G‐RNAs are secondary RNA species produced through the piRNA pathway (Seth et al., 2013; Wedeles et al., 2013). This pathway activates or maintains the expression of endogenous mRNAs in germline cells, protecting them from silencing by piRNAs. Therefore, this type of RNAa differs from the canonical RNAa observed in mammals in that the activating 22G‐RNAs interact with nascent mRNAs on the chromatin of their target genes rather than at the promoter region (Li, 2017).

In plants, non‐canonical RNAa has been shown, but there is currently no definitive evidence for a conserved mammalian‐type RITA complex. Instead, RNA‐directed DNA methylation is triggered by small dsRNAs derived from the expression of inverted repeat sequences that target a specific intron (intron 2) in the class‐C floral homeotic gene *pMADS3* in *Petunia* (Shibuya et al., 2009). This interaction results in DNA methylation at specific CGs within the intron, causing ectopic upregulation.

As noted above, the main components of the RITA complex in mammalian cells are AGO2, CTR9 and RHA proteins. While AGO2 is a conserved protein across living organisms (Swarts et al., 2014), BLASTp analysis was performed to identify potential homologues of the human CTR9 and RHA proteins in representative model insects from different orders. These were *Aedes aegypti*, *Drosophila melanogaster* (Diptera), *Bombyx mori* (Lepidoptera), *Tenebrio molitor* (Coleoptera) and *Acyrthosiphon pisum* (Hemiptera). Homologues of both genes were found in all species, with distances from *H. sapiens* ranging from 0.45 to 0.59 for CTR9 and 0.60 to 0.73 for RHA. The clustering of species was similar for both proteins, with *Ae. aegypti* and *D. melanogaster* clustering together, *B. mori* and *T. molitor* clustering together (for RHA), and *H. sapiens* and *Mus musculus* clustering together. The proteins from *A. pisum* were on separate branches from those of other insects (Figure 2).

**FIGURE 2:**
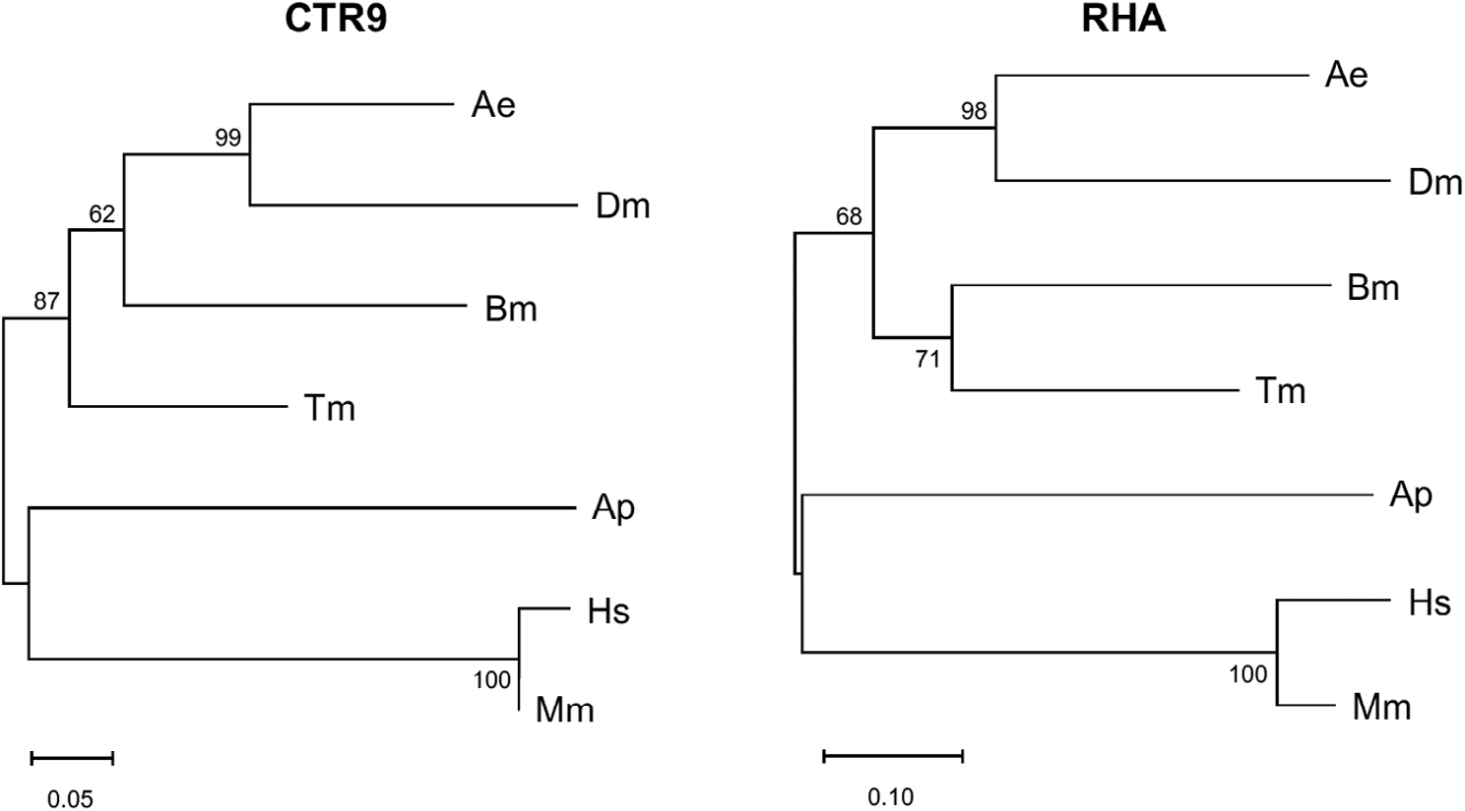
Phylogenetic tree of the amino acid sequences of CTR9 and RHA from representative model insects and their mammalian homologues. Maximum‐likelihood trees were constructed with 1000 bootstraps based on Muscle alignment in MEGA (https://www.megasoftware.net/). The percentage of replicate trees in which the associated taxa clustered together in the bootstrap test is shown next to the branches. Ae, *Aedes aegypti*; Dm, *Drosophila melanogaster*; Bm, *Bombyx mori*; Tm, *Tenebrio molitor*; Ap, *Acyrthosiphon pisum*; Hs, *Homo sapiens*; Mm, *Mus musculus*. The accession numbers for the RHA sequences were Ae (AAEL004859), Dm (NP_476641), Bm (NP_001093309), Tm (XP_068916745), Ap (XP_008183325), Hs (AAB48855), and Mm (NP_031868). The accession numbers for the CTR9 sequences were Ae (XP_021712620), Dm (NP_788448), Bm (XP_028040937), Tm (KAJ3627405), Ap (XP_001944071), Hs (NP_055448), and Mm (AAH10344).

## RNAa IN INSECTS

There are only a few examples of RNAa in insects. The first instance of RNAa in insects was reported from *Ae. aegypti* from our lab (De Hayr et al., 2020). This was achieved by targeting the promoters of endogenous mosquito genes using both dsRNA and saRNAs. About 500 bps long dsRNAs, up to 100 bp before the transcriptional start site (TSS) region, were designed for the promoter regions of *hsp70*, a housekeeping gene, and *Relish 1b* (*Rel1b*) and *Relish 2* (*Rel2*) as immune genes. The idea was to utilise the RNAi machinery to produce small RNAs that could function as saRNAs. Transfection of the dsRNAs into *Ae. aegypti* Aag2 cells resulted in significant increases in the expression of all three genes. Induction of *Rel1b* and *Rel2* led to increased expression of their downstream antimicrobial effector genes, *Gambicin*, *Defensin D*, *Defensin E* and *Cecropin A*. Additionally, dsRNAs targeting the promoter regions of other immune genes in the JAK–STAT pathway, *Dome* and *Hop*, successfully elevated the transcription levels of both genes in Aag2 cells (De Hayr et al., 2020).

Following the successful induction of the five genes with dsRNAs to their promoters, two 21 nt saRNAs were designed for the promoter region of *Dome* using an algorithm developed by Harris et al. (Harris et al., 2017). Transfection of the saRNAs resulted in 40%–50% induction of the gene, comparable to that of the dsRNA (De Hayr et al., 2020). While these inductions were in the Aag2 cell line, targeting the *Gambicin* promoter by injecting saRNAs into *Ae. aegypti* female mosquitoes also demonstrated successful induction of the gene in vivo (De Hayr et al., 2020).

In addition to endogenous promoters, RNAa can also be used to boost the expression of genes driven by exogenous promoters, such as plasmid expression systems, when transfected into host cells. This could be particularly useful in cases where the expression of the cloned gene is relatively weak. A dsRNA designed for the OpIE2 promoter in the pIZ/V5 vector induced GFP expression as a reporter in Aag2 and *Spodoptera frugiperda* (Sf9) cell lines (De Hayr et al., 2020). One of the saRNAs designed to the TSS did not induce GFP expression, emphasising the importance of the location of the designed saRNAs (see Box 1). When the seed region of one of these saRNAs was mutated, RNAa was abolished, consistent with studies in mammalian cells (Meng et al., 2016). In human cells, the 5′ long terminal repeat (5′LTR) promoter of the exogenous murine stem cell virus (MSCV), stably integrated into the human cancer cell line HEK293T via lentiviral infection, could be induced by saRNAs (Harris et al., 2017).

In multiple experiments conducted in *Ae. aegypti* and Sf9 cells, the control dsRNA or saRNA did not induce gene expression (De Hayr et al., 2020). Therefore, indirect activation of target genes by exogenous dsRNA (e.g., via the cGAS‐STING pathway) or by saRNA alone may not account for the specific gene induction. While the presence of a canonical RNAa process in insects requires further investigation, the involvement of AGO2 in RNAa and the presence of homologues of the main proteins in the RITA complex (CTR9 and RHA) in insects increase the likelihood of such a process.

In a recent study, antisense DNA oligos (ASOs) to the promoter region of a serine/threonine kinase (*STK*) gene in *Ae. aegypti* were shown to successfully induce expression of the gene in Aag2 cells (Hussain et al., 2025). This further reduces cost, given that DNA oligos are significantly cheaper than siRNAs or dsRNAs. While plain ASOs were used in the study mentioned, chemically modified oligonucleotides (e.g., phosphorothioate, locked nucleic acid [LNA] or 2′‐O‐methyl) are recommended for improved stability, target binding and reduced toxicity (Chan et al., 2006). Nevertheless, the mechanism of gene induction by ASOs remains unclear and unlikely to utilise the canonical RNAa mechanism. One potential mechanism is binding to and targeting the natural antisense transcript (NAT) of the target gene, resulting in derepression caused by the NAT (Jiao & Slack, 2014; Szukowska et al., 2025).

## RNAa IN TICKS

In arthropods, RNAa has also been demonstrated in ticks, although not through the canonical RNAa mechanism. Kwofie et al. showed that in the Asian longhorned tick vector, *Haemaphysalis longicornis*, the endochitinase‐like gene (*HlemCHT*) targeted by saRNA in the tick's eggs resulted in a 19‐fold upregulation of the gene, as assessed by qRT‐PCR (Kwofie et al., 2023). The increase in transcription coincided with early egg development and hatching. In this case, the investigators targeted the 3′‐UTR of *HlemCHT* with a 497 bp dsRNA, based on studies showing that RNAa can be induced by targeting 3′ non‐coding transcripts expressed from downstream of their 3′ termini (Yue et al., 2010). This study suggests RNAa might occur across Arthropoda.

## mi‐RNAa IN INSECTS

Endogenous miRNAs can potentially induce RNAa when they bind to the promoter region of a target gene. Although there are several examples from mammalian systems (e.g., Huang et al., 2012; Majid et al., 2010; Place et al., 2008), no experimental evidence exists to demonstrate mi‐RNAa effects in insects through interaction with a promoter. This may partly be due to limited research in this area and to the fact that most researchers focus on the negative interactions between miRNAs and their target genes (downregulation). However, several miRNAs have been shown to upregulate target gene expression. For instance, in *Locusta migratoria*, miR‐276 upregulates the transcription coactivator gene *brahma* (*brm*), which, in turn, promotes egg‐hatching synchrony in locusts (He et al., 2016). The mechanism involves increasing the stability of the target gene's mRNA by altering the *brm* mRNA's stem‐loop structure. A human blood miRNA, hsa‐miR‐21‐5p, was shown to induce the *vitellogenin* gene in *Ae. aegypti* mosquitoes (Perdomo et al., 2021). Additionally, in *Ae. aegypti*, aae‐miR‐275, significantly induced after blood feeding and essential for blood digestion, positively regulates its target gene, sarco/endoplasmic reticulum Ca^2+^ adenosine triphosphatase, by binding the 3′‐UTR of the target gene (Zhao et al., 2017).

## CONCLUDING REMARKS

RNAa represents a largely unexplored aspect of RNA regulation in insects. While RNAi has dominated the field, growing evidence suggests that small RNAs can also enhance transcription and influence epigenetic landscapes. By recognising RNAa as a potential tool, Insect Molecular Biology could adopt a versatile method for gene activation, functional genomics, and applied research. A systematic study of RNAa might ultimately broaden the RNA toolbox from silencing to precise, programmable activation.

RNAa could provide a powerful tool in basic and applied research, potentially leading to translational research. In basic research, RNAa could be used alongside RNAi and CRISPR‐based approaches as a gain‐of‐function tool. In particular, RNAa would activate endogenous genes transiently without altering the genomic sequence, which is especially valuable for studying essential genes whose upregulation or knockout could be lethal. Furthermore, when developmental regulatory genes are considered, their temporal activation patterns may be useful for life‐stage‐specific functional genomics studies. Similarly, RNAa could be used to modulate immunity, metabolism, behaviour or hormonal pathways without the need for genetic modification. Nevertheless, RNAa could also be incorporated into insect transgenesis or CRISPR for continuous expression or for regulation under inducible promoters, for example, induced upon blood feeding. This could be particularly valuable for upregulating immune or antiviral genes, thereby potentially reducing vector competence. This approach may be combined with symbiont‐based strategies, such as *Wolbachia*, to activate host pathways that influence symbiont density or pathogen interference. Furthermore, RNAa could be used to increase gene expression from transfected plasmids, particularly for genes with low expression or weak promoters.

So far, all examples of the translational applications of RNAa are from mammalian systems, with therapeutic applications in cancer, Alzheimer's disease and metabolic disorders. For instance, RAG‐01 and MTL‐CEBPA are saRNAs at phase I and phase II clinical trials for liver and bladder cancer, respectively (Hegde et al., 2026). MTL‐CEBPA, formulated in liposomal nanoparticles, targets the promoter of the transcription factor CCAAT/enhancer‐binding protein‐*α* (CEBPA) (Reebye et al., 2018). Induction of CEBPA significantly reverses fibrosis and steatosis, improving liver function. RAG‐01, which activates the *p21* gene, a cyclin‐dependent kinase inhibitor 1A gene, is being trialled for the treatment of non‐muscle‐invasive bladder cancer (Qian et al., 2025).

Several key questions remain regarding the application of RNAa in insects. We know that RNAi works better in some insect orders than in others (Zhu & Palli, 2020). Does RNAa operate broadly across insect orders, or is it restricted to certain taxa or cell types? Can RNAa be reliably programmed, or is it highly context‐dependent? How does RNAa interact with endogenous small RNA pathways? Are the canonical RNAa components involved in RNAa in insects? What is the specificity of RNAa and its off‐target effects? Addressing these questions will require systematic exploration, including cross‐species comparisons, dedicated RNAa screens, chromatin analyses, and so forth. Promoter annotation in insects, especially non‐model species, remains incomplete, making the rational design of promoter‐targeting RNAs more difficult. Finally, distinguishing true RNAa from indirect effects, such as stress responses or compensatory transcription, requires careful experimental design, including suitable controls and transcription‐level readouts.

## AUTHOR CONTRIBUTIONS

**Sassan Asgari:** Conceptualization; investigation; funding acquisition; writing – original draft; visualization; writing – review and editing.

## CONFLICT OF INTEREST STATEMENT

The author declares no competing interests.

BOX 1 **Considerations for designing saRNAs**

Designing saRNAs is not as straightforward as siRNAs. Based on the literature and trials, a number of suggestions are made here:

**1. saRNA sequence:** Design of saRNA duplexes follows similar rules to siRNA, considering they become associated with the AGO protein. They should typically be about 19 nt, contain 2 nt overhangs on the 3′‐ends, have a GC content of 40%–60%, and asymmetric thermodynamic stability in base‐pairing at the 5′‐end (Wang et al., 2015). In insects and other invertebrates, dsRNA can also be designed to target the promoter region, which is subsequently processed by the RNAi pathway and used as saRNAs (De Hayr et al., 2020). In addition to saRNAs, antisense DNA oligos could be utilised for gene induction (see RNAa in insects in the text).

**2. Target location:** saRNAs are typically designed to the promoter region of genes upstream of the transcription start site, with −100 to −1000 bp. Trials have shown that targets in the −200 to −500 bp region show optimal activation (Wang et al., 2015). It is more efficient to design at least two and up to six saRNAs in the promoter region to select the one with the highest activation effect on the target gene (Janowski & Corey, 2010).

**3. saRNA control**. Similar to RNAi experiments, a scrambled or mismatched saRNA is essential for inclusion in control treatments.

**4. Off‐target effects**. As with siRNAs, off‐target effects are possible. To avoid these effects, genome browser databases could be utilised to exclude potential off‐target sequences, considering both strands of the saRNA duplex when used for RNAa. Musiała‐Kierklo et al. demonstrated that 5′‐biotin modification on the sense strand could abolish its potential off‐target activity (Musiała‐Kierklo et al., 2025).

## Data Availability

Data sharing not applicable to this article as no datasets were generated or analysed during the current study.
